# New Delhi Metallo-β-Lactamase 4–producing *Escherichia coli* in Cameroon

**DOI:** 10.3201/eid1809.120011

**Published:** 2012-09

**Authors:** Laurent Dortet, Laurent Poirel, Nadia Anguel, Patrice Nordmann

**Affiliations:** Institut National de la Santé et de la Recherche Médicale Paris, France (L. Dortet, L. Poirel, P. Nordmann);; and Hôpital de Bicêtre–Assistance Publiques des Hôpitaux de Paris, Le Kremlin-Bicêtre, France (L. Dortet, N. Anguel, P. Nordmann)

**Keywords:** New Delhi metallo-β-lactamase, NDM variant, carbapenemase, Enterobacteriaceae, Escherichia coli, bacteria, Cameroon, Africa

**To the Editor:** The metallo-β-lactamase (MBL) group of enzymes inactivates many β-lactam antimicrobial drugs. First identified from a *Klebsiella pneumoniae* strain recovered from a patient hospitalized in India, the New Delhi metallo-β-lactamase-1 (NDM-1), particularly in *Enterobacteriaceae*, is now the focus of worldwide attention (*1*). Whereas India and Pakistan were considered as the main reservoirs of the *bla*_NDM-1_ gene (*2*) that produces this MBL, several NDM-1–producing *Enterobacteriaceae* isolates have been reported from the Balkan states and the Middle East, suggesting that those areas might be secondary reservoirs (*2*).

Since 2010, 3 NDM-1 point-mutation variants have been described (*3*–*5*). The first variant, NDM-2, was identified from an *Acinetobacter baumannii* isolate collected from a patient transferred from a hospital in Egypt to Germany (*4*). Subsequently, a clonal dissemination of NDM-2–producing *A. baumanni* was described in Israel (*6*). The second variant, NDM-4, which was identified in *Escherichia coli* from a patient hospitalized in India, possessed a higher carbapenemase activity compared with NDM-1 (*5*). The most recent variant, NDM-5, was identified in *E. coli* from a patient who had a history of hospitalization in India (*3*).

As recommended for the detection of carbapenemase producers (*7*), a rectal swab specimen was collected from a patient transferred from Cameroon to France. The *E. coli* strain FEK was isolated from the specimen. He had been hospitalized for 1 month in Douala for an inflammatory syndrome associated with a kidney failure before his transfer to Paris. No history of travel in India was reported for this patient. Susceptibility testing was performed by disk diffusion assay (Sanofi-Diagnostic Pasteur, Marnes-la-Coquette, France), and results were interpreted according to the updated guidelines of the Clinical and Laboratory Standards Institute (Wayne, PA, USA; www.clsi.org). The MICs were determined by using Etest (bioMérieux, La Balmes-Les-Grottes, France) on Mueller-Hinton agar at 37°C.

*E. coli* FEK was fully resistant to all β-lactam antimicrobial drugs, including imipenem, meropenem, ertapenem, and doripenem (MICs >32 mg/L for all carbapenems). This isolate was also resistant to aminoglycosides, except amikacin, and to fluoroquinolones. We performed PCR amplification followed by sequencing on whole-cell DNA, as described (*8*). We identified the *bla*_NDM-4_, *bla*_CTX-M-15_, and *bla*_OXA-1_ genes. *E. coli* FEK also harbored the *aacA4* gene encoding the AAC(6′)-Ib acetyltransferase that confers high-level resistance to aminoglycosides, except amikacin. Results of multilocus sequence typing analysis performed as described (*5*) showed that the isolate belonged to sequence type (ST) ST405. Identification of this ST type among NDM-producing *E. coli*, compared with NDM-4– and NDM-5–producing *E. coli* in ST648, demonstrated that the spread of NDM-4 occurred among unrelated *E. coli* clonal backgrounds (*3*,*5*).

Plasmid DNA of *E. coli* FEK was extracted and analyzed as described (*5*). A single, ≈120-kb plasmid was identified. Direct transfer of the β-lactam resistance marker into *E. coli* J53 was attempted by liquid mating-out assays at 37°C. With the exception of the aminoglycoside amikin, transconjugants from *E. coli* were resistant to β-lactam antimicrobial drugs. MICs of imipenem, meropenem, ertapenem, and doripenem were 6, 3, 6, and 4 mg/L, respectively. The transconjugants harbored an ≈120-kb plasmid carrying *bla*_NDM-4_ and the *bla*_CTX-M-15_, *bla*_OXA-1_, and *aacA4* genes. We performed PCR-based replicon typing as described (*5*) and showed that this *bla*_NDM-4_–positive plasmid belonged to the IncFIA incompatibility group. The IncF incompatibility group was previously reported to be associated with *bla*_NDM-4_ and *bla*_NDM-5_ (*3*,*5*).

By analyzing genetic structures surrounding the *bla*_NDM-4_ gene, performed by PCR mapping as described (*8*), we identified insertion sequence IS*Aba125* upstream and the bleomycin resistance gene *ble*_MBL_ downstream of the *bla*_NDM-4_ gene. The same genetic environment has been observed for most NDM-1–positive enterobacterial isolates (*8*). We showed in previous research that expression of *ble*_MBL_ conferred high-level resistance to bleomycin and bleomycin-like molecules (*9*); accordingly, the *E. coli* clinical isolate and its transconjugant were highly resistant to bleomycin (MIC >512 µg/mL) (*9*).

The patient had a history of Hodgkin lymphoma treated by 8 sessions of bleomycin chemotherapy 1 year before his hospitalization. This anticancer drug is widely distributed throughout the body following intravenous administration, and plasmatic concentrations increase in proportion with the increase of the dose (*10*). Because the patient was successively treated with 30 mg of bleomycin, the serum levels achieved (≈2–5 mg/mL) might have contributed to selection of the *ble*_MBL_ gene. Similarly, the multiple courses of antibacterial drug therapy administered in Cameroon (including carbapenems) could have contributed to selection of the *bla*_NDM-4_ gene.

By culturing rectal swab samples from the patient, we identified fecal carriage of *E. coli* carrying a plasmid-encoded *bla*_NDM-1_ gene. That strain had a distinct ST type (ST5) compared with the index strain. The plasmid carrying the *bla*_NDM-1_ gene with the *bla*_OXA-1_ and *aacA4* genes belonged to the IncFIA incompatibility group.

β-Lactamase NDM-4 displaying increased carbapenemase activity compared with NDM-1 was described in a patient hospitalized in India (*5*). This study shows that NDM-4 producers are also present in Africa; specifically, in the highly populated city of Douala, providing an environment that may promote the dissemination of those strains. We showed that the same patient was carrying strains expressing 2 NDM variants**,** possibly indicating ongoing evolution of NDM variants.
